# Hemoperitoneum caused by spontaneous rupture of hepatocellular carcinoma in noncirrhotic liver. A case report and systematic review

**DOI:** 10.1515/med-2020-0202

**Published:** 2020-08-03

**Authors:** Nicola Tartaglia, Alessandra Di Lascia, Pasquale Cianci, Alberto Fersini, Mario Pacilli, Giovanna Pavone, Antonio Ambrosi

**Affiliations:** Department of Medical and Surgical Sciences, University of Foggia, Luigi Pinto Street, No. 1, 71122, Foggia, Italy

**Keywords:** hepatocellular carcinoma, hemoperitoneum, noncirrhotic liver, transcatheter arterial embolization, staged hepatectomy

## Abstract

**Introduction:**

Hepatocellular carcinoma (HCC) is the sixth most common cancer. Spontaneous rupture of HCC is an acute complication with a high mortality rate. The HCC principally arises in the background of chronic liver disease and cirrhosis of the liver. In the last few years, the rising incidence of HCC in noncirrhotic liver suggests the presence of other factors that may play a role in liver carcinogenesis.

**Methods:**

We reviewed all cases treated at the University Surgical Department of Ospedali Riuniti of Foggia from 2009 to 2018. Only a single case of hemoperitoneum caused by spontaneous rupture of HCC in noncirrhotic liver was found. An extensive search of the relevant literature was carried out using MEDLINE, and a total of 58 published studies were screened from the sources listed.

**Conclusions:**

The management of this devastating emergency should be carefully analyzed, with stabilization of vital signs as soon as possible. Patient with ruptured HCC and hemoperitoneum without a prior history of cirrhosis and viral infections benefited from the role of transcatheter arterial embolization (TAE) as the preliminary treatment in order to have a more precise diagnosis and an optimal stabilization of the patient. Delayed or staged hepatectomy after TAE represents the definitive treatment.

## 1 Introduction

Hemoperitoneum due to nontraumatic liver injury can be caused by several different neoplastic and nonneoplastic diseases. Hepatocellular carcinoma (HCC) represents 90% of primary liver cancers, being the sixth most common cancer, and it is the second leading cause of cancer-related death [1,2]. Spontaneous rupture of HCC is an acute complication with a high mortality rate. Its incidence reached 8–26% in Asia and 3% in the United Kingdom. The mean age of patients with rupture was 75–79 years [3]. The HCC principally arises in the background of chronic liver disease and cirrhosis of the liver [4]. Most important causes for cirrhosis in patients with HCC are chronic hepatitis B, chronic hepatitis C virus (HCV), and alcoholic liver disease. Efficacious treatments for HCV have decreased the risk of progression to cirrhosis and development of HCC secondary to HCV but, in the last few years, the rising incidence of HCC in noncirrhotic liver suggests the presence of other factors that may play a role in liver carcinogenesis [5].

Actually, several treatments for the hemoperitoneum associated with the rupture of HCC: emergent hepatic resection, placation or packing, hepatic artery ligation, and transcatheter arterial embolization (TAE) are reported in the literature.

The aim of this study is to review the literature focusing on various treatments of hemoperitoneum in cases of spontaneous ruptured HCC on noncirrhotic liver and to investigate the outcomes of treatment and factors associated with prognosis considering different surgeons’s personal experience.

## 2 Materials and methods

From January 2009 to December 2018, about 45 cases of hemoperitoneum were performed at the University Surgical Department of Ospedali Riuniti of Foggia. Cases undergoing surgical treatment were spleen rupture, hemorrhagic diverticulitis, trauma from gunshot wounds or cutting weapons, bleeding neoplastic lesions, and treatments dealing with traditional or minimally invasive techniques (laparoscopic or robotic). We can report only a single case of hemoperitoneum caused by spontaneous rupture of hepatocellular carcinoma in noncirrhotic liver.

### 2.1 Literature review

An extensive search for relevant literature was carried out using MEDLINE (PubMed). The keywords used for the search were “spontaneous rupture of hepatocellular carcinoma,” hemoperitoneum rupture of HCC. Exclusion criteria were bleeding from metastasis, HCC on cirrhotic liver, adenomas, angiomas, and HCC due to HCV or HBV infection. Additional articles were identified by a manual search of the references from the key articles. Languages were restricted to English. We included articles from 1999.


**Ethical approval**: Not applicable.
**Consent for publication:** Written informed consent for publication of clinical data and clinical images was obtained from the patient.

## 3 Results

A total of 58 published studies were screened from the sources listed. After an accurate examination of all titles and text contents, 48 papers were excluded: 13 as not relevant, in six studies because there isn’t a mention about the presence of cirrhosis, six articles report only cases with a cirrhotic liver, four with a history of HBV infection, and two with HCV infection. Among the remaining, three studies were in other languages and 14 were excluded, as they were prior 1999.

Finally, 10 studies were included in the present study (Table 1). We introduced our case in the analysis. A total of 25 patients were analyzed.

**Table 1:** Literature review and our case

Author	Article type	No. of total cases	No. of noncirrhotic livers	Treatment
Ozen et al. [6]	Case report	01	01	Surgery
Yang et al. [7]	Case series	162	09	NA (not applicable)
Park et al. [8]	Case report	01	01	Surgery
Rossetto et al. [9]	Case report	01	01	Surgery → TACE
Bassi [10]	Case series	16	01	NA
Recordare et al. [11]	Case series	11	01	NA
Marini et al. [12]	Case series	13	03	NA
Vergara et al. [13]	Case series	06	01	NA
Descottes et al. [14]	Case series	22	05	Na
Kosaka et al. [15]	Case report	01	01	Surgery

NA, not applicable; surgery (surgical resection); TACE, transarterial chemoembolization.

### 3.1 Case report

A male patient, 75 years old, was urgently transported to the Emergency Room, due to onset of abdominal pain approximately 1 h before, associated with hypotension.

From the patient’s personal history: central obesity, arterial hypertension, treated with olmesartan medoxomil, diabetes mellitus 2, treated with repaglinide and glargine insulin, and cardioaspirin for prevention. Previous surgical interventions: open cholecystectomy at age 30, repair of epigastric hernia, and open prostatectomy for adenocarcinoma.

At the entrance, the patient’s parameters were, blood pressure 60/40 mmHg, 115 bpm heart rate, conserved diuresis, normochromic urine, and mild mental confusion (Shock Class III) 12.2 Hb at the first blood count and 10.4 on the second examination after an hour. On physical examination, the patient appeared pale and suffering. On palpation of the abdomen, it causes pain to the upper quadrants and the right lumbar.

**Figure 1:**
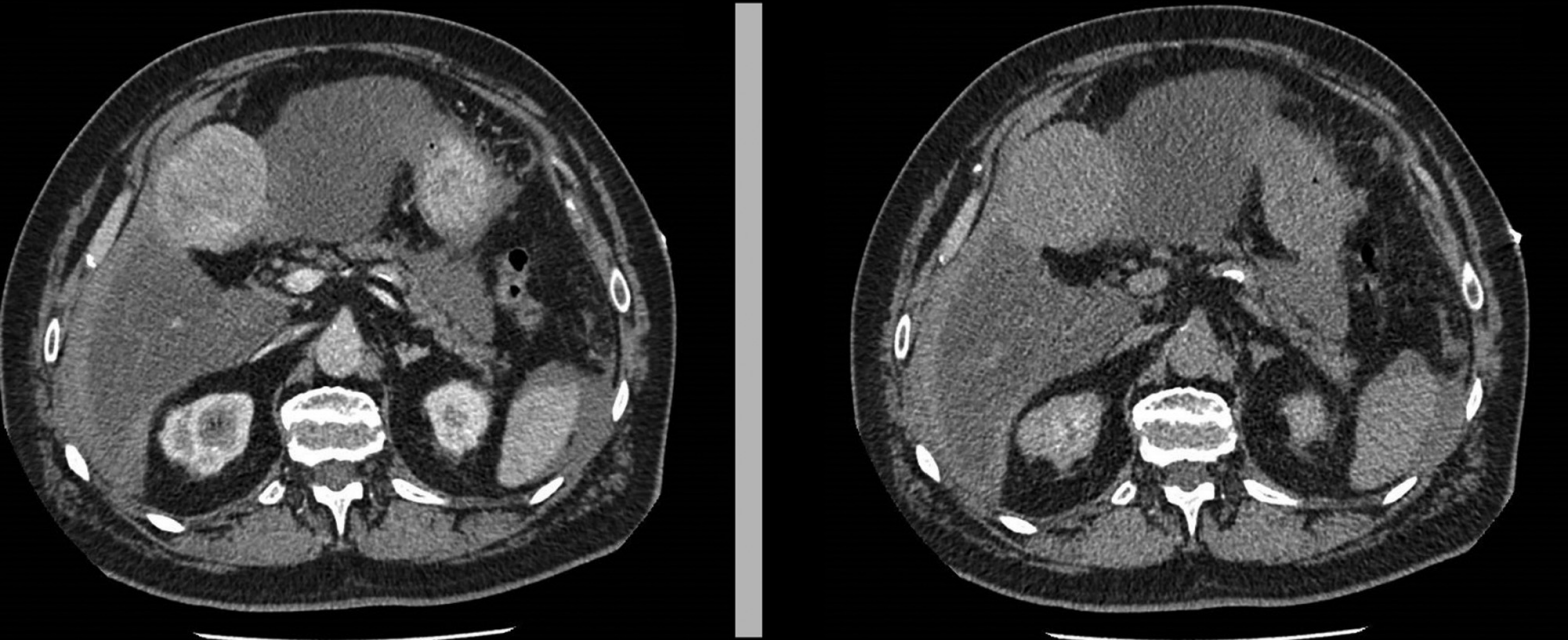
Neoformation of the IV hepatic segment.

An urgent ultrasonographic examination was performed, which showed fluid in the peritoneal cavity and an unidentified hepatic neoformation without other relevant findings. After administration of fluids and two units of leukodeplete blood cells, the vital parameters normalized and the patient was stabilized. A chest abdomen CT with contrast was required (Figure 1).

The TC images showed an oval neoformation with clear margins endowed with inhomogeneous enhancement, partly exophytic, at the level of the IV hepatic segment, with dimensions of 67 × 56 × 58 mm. The finding was not of unequivocal interpretation, therefore it had to be correlated to the biohumoral and clinical panel. Perihepatic and perisplenic blood effusion was also reported, but in the absence of active contrast spills. The liver appeared as enlarged as for chronic but not cirrhotic epatopathy, with the caliber of the inferior vena cava reduced. There are no other noteworthy findings.

The patient was therefore transferred to our Surgery Department. Set the continuous monitoring of vital parameters, the patient initially appeared stable, until the reappearance of signs of anemization, for which a new hemochromocytometric test was carried out, which showed hemoglobin values equal to 7.5 g/dL. Further transfusion of two units of red blood cells was carried out, and an urgent angiographic examination was required.

**Figure 2:**
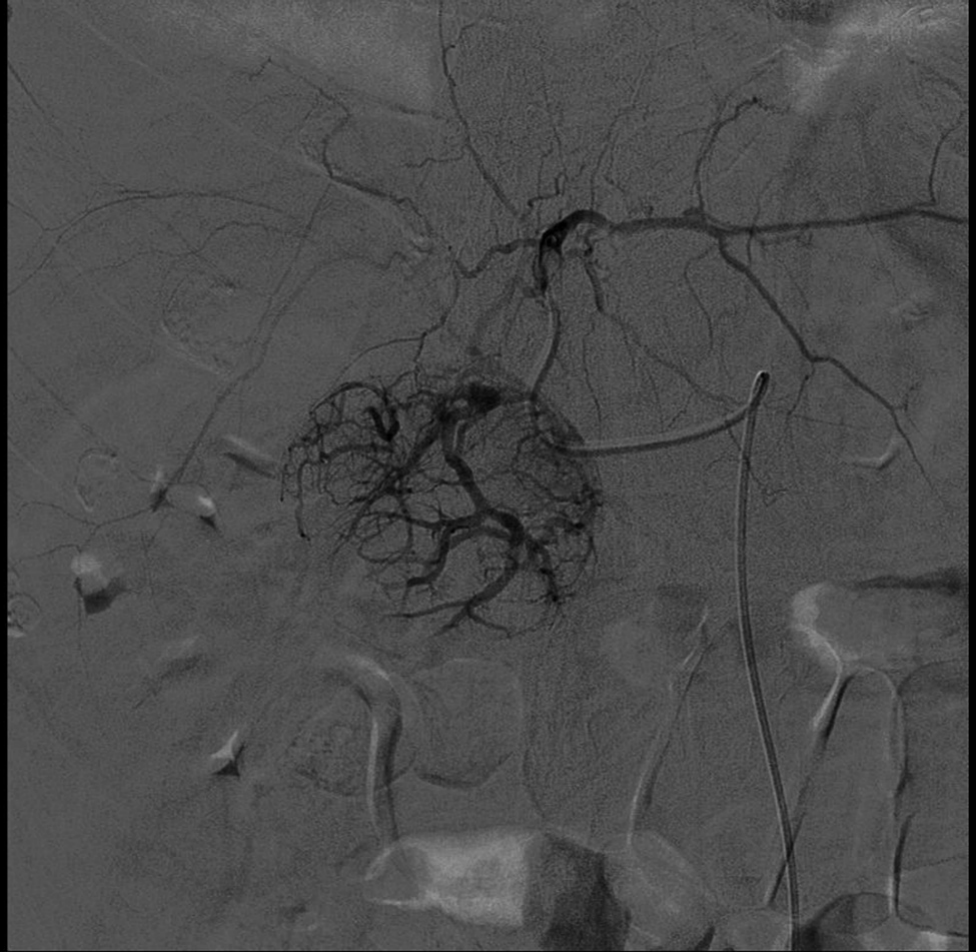
Expansive lesion of the IV hepatic segment.

The angiographic examination was performed, with right transfemoral arterial approach, and showed an expansive lesion of the IV hepatic segment, a source of active intra-abdominal bleeding (Figure 2).

**Figure 3:**
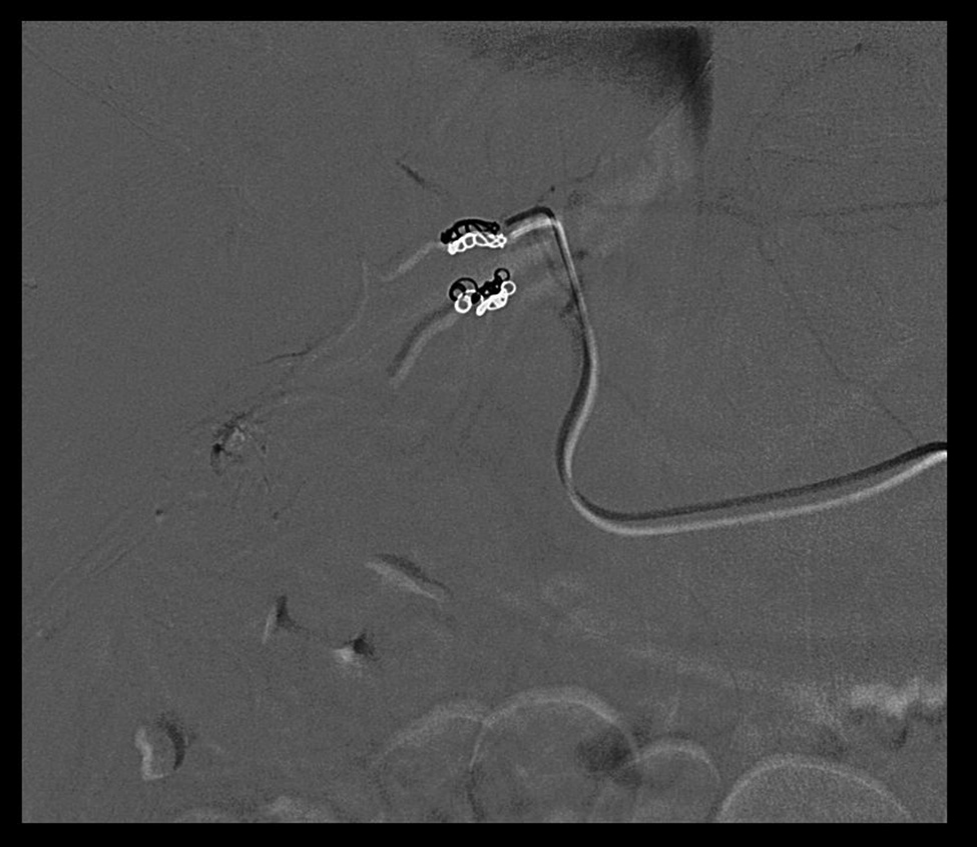
Metal microspirals and devascularizated lesion.

The arterial afferents of this lesion are embolized and metal microspirals are positioned, so that at the final check the lesion appears to be very well devascularized (Figure 3).

After angiography, the patient remained in stable condition. At this point, it was possible to complete the diagnostic and preoperative investigations in accordance with the EASL guide for the management of the HCC.

The serum AFP levels were normal, and in the same way all the other neoplastic markers. The patient was fully active, able to perform all pre-illness benefits without restrictions, therefore with a grade 0 of Performance Status according to the scale developed by the Eastern Cooperative Oncology Group. Child Pugh score was A5, as there was no presence of jaundice, ascites, or hepatic encephalopathy, while the albumin (3.8 g/dL) and PT (1.15) levels were preserved.

In liver surgery, the therapeutic strategy requires a careful preoperative staging, based on the BCLC staging system. In this case, the parameters we evaluated found a stage A, so the patient was the candidate for surgical resection of the tumor.

A laparotomy was performed because of pregress abdominal surgery. It revealed about 500 cc hemoperitoneum and a 6.5 cm diameter liver tumor, which was exophytic, ulcerated, and hemorrhagic. The tumor occupied the lower board of IV hepatic segment. The liver was not cirrhotic and apparently slightly increased in volume. A wedge resection of the IV hepatic segment was performed and the tumor was completely removed. The parenchyma was then controlled with bipolar forceps.

A subhepatic drain was placed. On the 2nd postoperative day, peristalsis reappeared, and the patient resumed feeding. No postoperative complications (ascites, hepatic failure, and pleural effusion) were detected, and on the 4th postoperative day the drain was removed and on the fifth the patient was discharged in general health.

Histology revealed HCC and no presence of cirrhosis.

## 4 Discussion

Hemoperitoneum is a possible scenario resulting in spontaneous HCC rupture. It has a prevalence of 1% in Western countries [16,17], and it occurs more frequently in cirrhotic liver [18,19].

Some conditions may increase the risk of intra-abdominal hemorrhage, as in the case of an inappropriate anti-aggregating or anticoagulant therapy, the presence of coagulopathy and thrombocytopenia associated with liver cirrhosis, or in the case of metastases and tumors with a diameter greater than 5 cm, as in the case of our study [20,21,22].

The main symptoms were abdominal tenderness, acute blood loss, and shock. Other symptoms and signs were distended abdomen, hypotension, abdominal tenderness, and tachycardia. It could happen that the tumor ruptures in a biliary tract, so a characteristic picture known as the Quincke triad in which there is hemobilia is formed, which causes melena associated with jaundice and abdominal pain.

All intra-abdominal bleeding cases represent a life-threatening condition. For this reason, the first step is certainly the stabilization of the patient and of his vital parameters with a close monitoring of the hemodynamic values [23,24].

Preoperative diagnosis of HCC rupture is difficult in patients without a previous history of cirrhosis or HCC. Abdominal sonography and CT scan are useful to demonstrate the presence of hemoperitoneum and liver tumors. An ultrasound examination should always be carried out, especially in the case of hemodynamic instability. The suspicion of hemoperitoneum can thus be confirmed and a CT examination can be performed for a more precise diagnosis [16].

Angiography is rarely performed for initial diagnosis because CT angiography makes it much better (88% vs 18%) [25,26] and permits conception of the entire gastrointestinal arterial system at the same time. However, the angiographic examination must always be evaluated, as it offers a valid alternative to the treatment of hemorrhage, thanks to the TAE [27,28], which can be a unique treatment or allow to resolve active bleeding, stabilize the patient, and bring him to the best conditions for surgery. Most postoperative deaths after emergency liver surgery are truly due to liver or hemodynamic failure [29,30], which is not seen after elective surgery. Moreover, delaying surgery gives time to achieve an etiologic workup and assess the patient in order to choose the best procedure for the individual patient.

TAE may reach successful hemostasis for ruptured HCC in up to 99% of the cases and has largely replaced other surgical hemostatic methods, such as ligation of the hepatic artery, perihepatic packing, and plication [31,32].

While using TAE exclusively, ruptured HCC still gives rise to the risk of peritoneal metastasis as a result of dissemination of cancer cell debris [33]. The therapeutic effect of the TAE is apparently inferior to hepatectomy and (Li et al.) [31,32,33,34] suggested that staged hepatectomy should follow the TAE in selected patients.

In the articles considered in our study, in almost all cases the treatment was exclusively surgical. In only one case, the treatment was first surgical and then followed by TACE due to local recurrence of the disease. Yang and Marini also included TAE treatments among their cases without mention of surgical retreatment. In this study, we would like to focus on how important the precise preoperative diagnosis and optimal patient stabilization are. For this reason, it was decided to adopt a strategy of ‘staged hepatectomy,’ in order to take advantage of the diagnostic therapeutic role of the angiographic examination with embolization. In fact, in this way it was possible to obtain a second imaging evaluation, after the CT examination, since it was a noncirrhotic liver, in a patient who was HCV and HBV negative, and in whom the diagnosis of HCC was more difficult. Furthermore, it was possible to devascularize the lesion, in order to prevent further bleeding and to allow an optimal preoperative stabilization of the patient.

## 5 Conclusions

Patients with ruptured HCC always arrive in the Emergency Room with an acute abdomen. With the availability of imaging studies and laboratory analyses, a rapid diagnosis is possible, but in some cases it is still a challenge, as in the case of HCC on noncirrhotic liver. The management of this devastating emergency should be closely scrutinized, with stabilization of vital signs and maintenance of hepatic perfusion as soon as possible. From the present study, a patient with ruptured HCC and hemoperitoneum without a prior history of cirrhosis and viral infections benefited from the role of TAE as the preliminary treatment in order to have a more precise diagnosis and an optimal stabilization of the patient. Delayed or staged hepatectomy after TAE represents the definitive treatment and had the highest survival rate (Shimada et al. and Miyamoto et al.) [4,25,34] and should be the procedure of choice for treatment of a spontaneously ruptured HCC.

## List of abbreviations

HCC: Hepatocellular carcinoma
TAE: Transarterial embolization
HCV: Hepatitis C virus
HBV: Hepatitis B virus
ECOG: Eastern cooperative oncology group
TACE: Transarterial chemoembolization

## References

[1] Ferlay J, Shin H, Bray F, Forman D, Mathers C, Parkin D. Estimates of worldwide burden of cancer in 2008: GLOBOCAN 2008. Int J Cancer. 2010;127(12):2893–917. 10.1002/ijc.25516. 21351269

[2] Cianci P, Tartaglia N, Altamura A, Fersini A, Sanguedolce F, Ambrosi A, et al. Hemoperitoneum due to breaking uterine adenosarcoma located in the omentum. Report of a case. Ann Ital Chir. 2016 Dec 20;5:S2239253X16026311. 28003568

[3] Buczkowski A, Kim P, Ho S, Schaeffer D, Lee S, Owen D, et al. Multidisciplinary management of ruptured hepatocellular carcinoma. J Gastrointest Surg. 2006;10(3):379–86. 10.1016/j.gassur.2005.10.012. 16504883

[4] El-Serag H, Rudolph K. Hepatocellular carcinoma: epidemiology and molecular carcinogenesis. Gastroenterology. 2007;132(7):2557–76. 10.1053/j.gastro.2007.04.061. 17570226

[5] Michelotti G, Machado M, Diehl A. NAFLD, NASH and liver cancer. Nat Rev Gastroenterol Hepatol. 2013;10(11):656–65. 10.1038/nrgastro.2013.183. 24080776

[6] Özen Ö, Tosun A, Akgül Ç. Spontaneous rupture of multifocal hepatocellular carcinoma: case report. Int Med Case Rep J. 2015;8:165–7. 10.2147/imcrj.s87746. PMC4544813 26316825

[7] Yang H, Chen K, Wei Y, Liu F, Li H, Zhou Z, et al. Treatment of spontaneous ruptured hepatocellular carcinoma: a single-center study. Pak J Med Sci. 2014;30(3):472–6. 10.12669/pjms.303.4001. PMC4048488 24948961

[8] Park K, Yang S, Yoon M. One stage resection of spontaneous rupture of hepatocellular carcinoma in the triangular ligament with diaphragm invasion: case report and review of the literature. World J Emerg Surg. 2012;7(1):30. 10.1186/1749-7922-7-30. PMC3544608 22995633

[9] Rossetto A, Adani G, Risaliti A, Baccarani U, Bresadola V, Lorenzin D, et al. Combined approach for spontaneous rupture of hepatocellular carcinoma. World J Hepatol. 2010;2(1):49. 10.4254/wjh.v2.i1.49. PMC2999262 21160956

[10] Bassi N. Management of ruptured hepatocellular carcinoma: Implications for therapy. World J Gastroenterol. 2010;16(10):1221. 10.3748/wjg.v16.i10.1221. PMC2839174 20222165

[11] Recordare A, Bonariol L, Caratozzolo E, Callegari F, Bruno G, Di Paola F, et al. Management of spontaneousbleeding due to hepatocellular carcinoma. Minerva Chir. 2002;57(3):347–56. 12029230

[12] Marini P, Vilgrain V, Belghiti J. Management of spontaneous rupture of liver tumours. Digest Surg. 2002;19(2):109–13. 10.1159/000052022. 11978996

[13] Vergara V, Muratore A, Bouzari H, Polastri R, Ferrero A, Galatola G, et al. Spontaneous rupture of hepatocelluar carcinoma: surgical resection and long-term survival. Eur J Surg Oncol. 2000;26(8):770–2. 10.1053/ejso.2000.1001. 11087643

[14] Descottes B, Lachachi F, Valleix D, Durand-Fontanier S, Sodji M, Pech de Laclause B, et al. Ruptured hepatocarcinoma. Report of 22 cases. Chirurgie. 1999;124(6):618–25. 10.1016/s0001-4001(99)00086-0 10676022

[15] Kosaka A, Hayakawa H, Kusagawa M, Takahashi H, Okamura K, Mizumoto R, et al. Successful surgical treatment for implanted intraperitoneal metastases of ruptured small hepatocellular carcinoma: report of a case. Surg Today. 1999;29(5):453–7. 10.1007/bf02483040. 10333419

[16] Battula N, Tsapralis D, Takhar A, Coldham C, Mayer D, Isaac J, et al. Aetio-pathogenesis and the management of spontaneous liver bleeding in the West: a 16-year single centre experience. HPB. 2012;14:382–9. 10.1111/j.1477-2574.2012.00460.x PMC3384862 22568414

[17] Sanguedolce F, Landriscina M, Ambrosi A, Tartaglia N, Cianci P, Di Millo M, et al. Bladder metastases from breast cancer: managing the unexpected. A systematic review. Urol Int. 2018;101(2):125–31. 10.1159/000481576. 29055945

[18] Tan FLS, Tan YM, Chung AYF, Cheow PC, Chow PKH, Ooi LL. Factors affecting early mortality in spontaneous rupture of hepatocellular carcinoma. ANZ J Surg. 2006;76:448–52. 10.1111/j.1445-2197.2006.03750.x 16768766

[19] Tartaglia N, Cianci P, Di Lascia A, Fersini A, Ambrosi A, Neri V. Laparoscopic antegrade cholecystectomy: a standard procedure? Open Med. 2016;11:429–32. 10.1515/med-2016-0078. PMC5329865 28352832

[20] Mounajjed T, Wu TT. Telangiectatic variant of hepatic adenoma: clinicopathologic features and correlation between liver needle biopsy and resection. Am J Surg Pathol. 2011;35:1356–63. 10.1097/PAS.0b013e31822280f3 21836491

[21] Dokmak S, Paradis V, Vilgrain V, Sauvanet A, Farges O, Valla D, et al. A single-center surgical experience of 122 patients with single and multiple hepatocellular adenomas. Gastroenterology. 2009;137:1698–705. 10.1053/j.gastro.2009.07.061 19664629

[22] Tartaglia N, Di Lascia A, Cianci P, Fersini A, Sanguedolce F, Iadarola R, et al. One stage surgery for synchronous liver metastasis from a neuroendocrine tumor of the colon. A case report. Ann Ital Chir. 2017 Nov 20;6:S2239253X17027694. 29176078

[23] Marini P, Vilgrain V, Belghiti J. Management of spontaneous rupture of liver tumours. Dig Surg. 2002;19:109–13. 10.1159/000052022 11978996

[24] Kirikoshi H, Saito S, Yoneda M, Fujita K, Mawatari H, Uchiyama T, et al. Outcomes and factors influencing survival in cirrhotic cases with spontaneous rupture of hepatocellular carcinoma: a multicenter study. BMC Gastroenterol. 2009;9:29. 10.1186/1471-230X-9-29 PMC2685387 19405938

[25] Ngan HH, Tso WKW, Lai CLC, Fan STS. The role of hepatic arterial embolization in the treatment of spontaneous rupture of hepatocellular carcinoma. Clin Radiol. 1998;53:338–41. 10.1016/s0009-9260(98)80004-4 9630270

[26] Cianci P, Fersini A, Tartaglia N, Ambrosi A, Neri V. Are there differences between the right and left laparoscopic adrenalectomy? Our experience. Annali Italiani Di Chirurgia. 2016;87:242–6. 27345193

[27] Liu CL, Fan ST, Lo CM, Tso WK, Poon RT, Lam CM, et al. Management of spontaneous rupture of hepatocellular carcinoma: single-center experience. J Clin Oncol. 2001;19:3725–32. 10.1200/JCO.2001.19.17.3725 11533094

[28] Neri V, Ambrosi A, Fersini A, Tartaglia N, Cianci P, Lapolla F, et al. Laparoscopic cholecystectomy: evaluation of liver function tests. Annali Italiani Di Chirurgia. 2014;85(5):431–7. 25601366

[29] Lai ECH, Lau WY. Spontaneous rupture of hepatocellular carcinoma: a systematic review. Arch Surg. 2006;141:191–8. 10.1001/archsurg.141.2.191 16490898

[30] Neri V, Ambrosi A, Fersini A, Tartaglia N, Lapolla F. Common bile duct lithiasis: therapeutic approach. Annali Italiani Di Chirurgia. 2013;84(4):405–10. 23917151

[31] Li WH, Cheuk EC, Kowk PC, Cheung MT. Survival after transarterial embolization for spontaneous ruptured hepatocellular carcinoma. J Hepatobil Pancreat Surg. 2009;16:508–12. 10.1007/s00534-009-0094-6 19381430

[32] Kung CT, Liu BM, Ng SH, Lee TY, Cheng YF, Chen MC, et al. Transcatheter arterial embolization in the emergency department for hemodynamic instability due to ruptured hepatocellular carcinoma: analysis of 167 cases. AJR Am J Roentgenol Rad Ther. 2008;191:231–9. 10.2214/AJR.07.3983 19020209

[33] Sonoda T, Kanematsu T, Takenaka K, Sugimachi K. Ruptured hepatocellular carcinoma evokes risk of implanted metastases. J Surg Oncol. 1989;41:183–6. 10.1002/jso.2930410310 2545975

[34] Shimada R, Imamura H, Makuuchi M, Soeda J, Kobayashi A, Noike T, et al. Staged hepatectomy after emergency transcatheter arterial embolization for ruptured hepatocellular carcinoma. Surgery. 1998;124:526–35. 9736905

